# Repulsive Environment Attenuation during Adult Mouse Optic Nerve Regeneration

**DOI:** 10.1155/2018/5851914

**Published:** 2018-09-12

**Authors:** Camila Oliveira Goulart, Henrique Rocha Mendonça, Julia Teixeira Oliveira, Laura Maria Savoldi, Luiza dos Santos Heringer, Alexandre dos Santos Rodrigues, Roberto Paes-de-Carvalho, Ana Maria Blanco Martinez

**Affiliations:** ^1^Laboratório de Neurodegeneração e Reparo, Departamento de Patologia, Programa de Pós-graduação em Patologia, Faculdade de Medicina, HUCFF, UFRJ, Rio de Janeiro, RJ, Brazil; ^2^Pólo Universitário Macaé, Unidade Integrada de Pesquisa em Produtos Bioativos e Biociências, UFRJ, Macaé, RJ, Brazil; ^3^Departamento de Neurobiologia, Programa de Pós Graduação em Neurociências, UFF, Niterói, RJ, Brazil

## Abstract

The regenerative capacity of CNS tracts has ever been a great hurdle to regenerative medicine. Although recent studies have described strategies to stimulate retinal ganglion cells (RGCs) to regenerate axons through the optic nerve, it still remains to be elucidated how these therapies modulate the inhibitory environment of CNS. Thus, the present work investigated the environmental content of the repulsive axon guidance cues, such as Sema3D and its receptors, myelin debris, and astrogliosis, within the regenerating optic nerve of mice submitted to intraocular inflammation + cAMP combined to conditional deletion of PTEN in RGC after optic nerve crush. We show here that treatment was able to promote axonal regeneration through the optic nerve and reach visual targets at twelve weeks after injury. The Regenerating group presented reduced MBP levels, increased microglia/macrophage number, and reduced astrocyte reactivity and CSPG content following optic nerve injury. In addition, Sema3D content and its receptors are reduced in the Regenerating group. Together, our results provide, for the first time, evidence that several regenerative repulsive signals are reduced in regenerating optic nerve fibers following a combined therapy. Therefore, the treatment used made the CNS microenvironment more permissive to regeneration.

## 1. Introduction

The regenerative capacity of CNS tracts has ever been a great hurdle to regenerative medicine. Only in the last 40 years, CNS regeneration was considered as feasible, with the focus of research on identifying and overcoming the inhibitory nature of CNS to axon growth. From 1980s until early 2000s, it was shown that central axons of the optic nerve were able to regenerate and reinnervate targets when a peripheral nerve bridge was grafted to form a passage where axons could avoid the degenerating central nervous tissue, partially restoring function [1–5]. Additional research by several groups showed that inhibition or degradation of repulsive guidance signals, such as SemaA3 family members, astrocyte-derived chondroitin sulfate proteoglycans (CSPG), and myelin-derived inhibitors such as NOGO-A, oligodendrocyte myelin glycoprotein (OMgp), or myelin-associated glycoprotein (MAG) allow regeneration within CNS paths [6–8]. However, the regeneration achieved by these approaches was not enough to restore function.

During the last 20 years, the research focus has changed to the enhancement of the intrinsic capacity of central neurons to elongate lesioned axons. Approaches that stimulate JAK-STAT and mTOR signaling, such as inflammatory stimuli or genetic deletions of PTEN and/or SOCS3, promoted pronounced regeneration within the optic nerve [9–11], and when these approaches were combined, there was a partial restoration of the visual function [12].

The stimulation of the intrinsic capacity of neurons to regenerate axons can benefit from environmental reduction/inhibition of repulsive guidance cues. Indeed, inflammatory-stimulated retinal ganglion cells regenerate longer distances when NOGO-A is counteracted [13]. Besides, the regenerative capacity of sciatic nerve axons to regenerate is abolished when axons reach an optic nerve graft [14], suggesting that an inhibitory environment prevents the regeneration of intrinsically growth-capable axons. If therapies that target the intrinsic growth capacity of axons also modulates the inhibitory environment of CNS remains to be elucidated.

Thus, the present work investigated the environmental content of the repulsive axon guidance cues, such as Sema3D and its receptors, myelin debris, and astrogliosis, within the regenerating optic nerve of mice submitted to intraocular inflammation + cAMP combined to conditional deletion of PTEN in RGC after optic nerve crush.

## 2. Methods

### 2.1. Surgical Procedure

Optic nerve surgery and intraocular injections were performed as previously described [12, 15]. Briefly, to delete pten gene in RGC, pten^flx/flx^ in a C57BL/6 background mice received an intravitreal injection (3 *μ*L) of an adeno-associated virus (AAV2, 10^12^ GC/mL) expressing Cre recombinase (AAV2-Cre under the control of the CMV promoter; Vector Laboratories) (Regenerating group, *n* = 5), as shown to be effective by Kwon et al. [7]. Two weeks after virus injection, the left optic nerve was exposed and crushed with fine forceps (Dumont; WPI) for 5 s. To increase regeneration, zymosan (Sigma; 12.5 *μ*g/*μ*L, sterilized before use) was injected, along with the cAMP analog CPT-cAMP (Sigma; 50 *μ*M, 3 *μ*L), into the posterior chamber of the eye, immediately after surgery. As a control, C57BL/6 mice just had the left optic nerve exposed and crushed for 5 s (Crush-only group, *n* = 5). Before the end of each survival time, animals were injected intraocularly with cholera toxin B fragment (CTB) to trace retinal projections. To all the procedures, mice were anesthetized with either ketamine or xylazine and immobilized in a stereotaxic head holder.

### 2.2. Tissue Preparation

Two weeks after surgery, mice were perfused transcardially with 4% phosphate-buffered paraformaldehyde (PFA). Optic nerves were carefully dissected, placed in 4% PFA for 1 h, and transferred to 20% sucrose in 0.1 M PBS at 4°C overnight followed by 30% sucrose overnight at 4°C. Optic nerves were then embedded in OCT (Tissue Tek Medium), frozen, and cryostat-sectioned longitudinally at 14 *μ*m thick. Nerve sections were placed on 1% bovine gelatin-coated coverslips and stored at 20°C until used.

In order to analyze central projections, mice were maintained until twelve weeks of survival time and then perfused transcardially with 4% phosphate-buffered paraformaldehyde (PFA). Brains were carefully dissected, placed in 4% PFA for 1 h, and transferred to 20% sucrose in 0.1 M PBS at 4°C overnight followed by 30% sucrose overnight at 4°C. Brains were also embedded in OCT (Tissue Tek Medium), frozen, and sectioned at 40 *μ*m in the coronal plane between the rostral diencephalon and caudal mesencephalon. Sections were maintained in 0.1 M PBS and stored at 4°C until used.

### 2.3. WFA Histochemistry

Sections were washed three times in 0.1 M PBS (pH 7.4), blocked (PBS containing 0.3% Triton X-100 and 10% normal donkey serum) at room temperature for 1 h, then were incubated in fluorescein Wisteria floribunda lectin (WFA, 1 : 100, Vector) for 2 h at room temperature. Next, the slices were counterstained with DAPI (Molecular Probes) for 5 min and coverslipped with fluoromount (Sigma) for visualization. Staining was analyzed and documented on a laser scanning microscope (LSM 510 META, Zeiss, Germany).

### 2.4. Immunofluorescence Labeling

Sections were washed three times in 0.1 M PBS (pH 7.4), blocked (PBS containing 0.3% Triton X-100 and 10% normal donkey serum) at room temperature for 1 h, then were incubated overnight in one or more of the following antibodies: rabbit monoclonal anti-CTB (1 : 500, GenWay), rat polyclonal anti-MBP (myelin basic protein, 1 : 200, Millipore), mouse monoclonal anti-CC1 (1 : 200, Abcam), rabbit polyclonal anti-IBA1 (1 : 250, Wako), rabbit monoclonal anti-TMEM119 (1 : 100, Abcam), and rabbit polyclonal anti-GFAP (glial fibrillary acidic protein, 1 : 100, Sigma). Sections were washed three times in PBS, incubated in the appropriate secondary antibodies (Alexa-fluor 488 Donkey anti-rabbit, Alexa-fluor 594 Donkey anti-rabbit, Alexa-fluor 488 Donkey anti-mouse, and Alexa-fluor 488 Goat anti-rat, 1 : 500, Molecular Probes) for 2 h at room temperature, counterstained with DAPI (Molecular Probes) for 5 min, and coverslipped with fluoromount (Sigma) for visualization. Negative control sections were processed identically except that the primary antibodies were omitted. Staining was analyzed and documented on a laser scanning microscope (LSM 510 META, Zeiss, Germany).

Quantification of histochemistry and immunofluorescence data was performed using ImageJ by determining immunostained densities and the number of immuno-positive cells [16]. Cell counts were performed identifying DAPI-positive nuclei surrounded by Iba-1 or TMEM119 staining under the microscope. One field at 20x magnification, at a fixed distance of 1.5 mm from the injury site in longitudinal sections of the nerve, was analyzed from five animals per group.

### 2.5. Electron Microscopy

To observe myelin debris, transmission electron microscopy was performed as described in [17]. Briefly, mice were sacrificed two weeks after injury with an overdose of ketamine/xylazine and perfused transcardially with 4% paraformaldehyde + 2% glutaraldehyde in 0.1 M phosphate buffer pH 7.4. 1 mm long segments of the optic nerve 1.5 mm distal to the lesion site were immersion-fixed in 2.5% glutaraldehyde for 2 h, washed in 0.1 M cacodylate buffer (pH 7.4), and postfixed for 1 h in 1% (wt/vol) osmium tetroxide containing 0.8% potassium ferrocyanide and 5 nM calcium chloride in 0.1 M cacodylate buffer (pH 7.4). Segments were washed in 0.1 M cacodylate buffer (pH 7.4) and distilled water and stained in 1% (wt/vol) uranyl acetate overnight, dehydrated in graded acetone, infiltrated with Poly/Bed 812 resin (Polysciences), and polymerized at 60° for 48 h. Ultrathin cross sections (70 nm) were collected on copper grids and contrasted in uranyl acetate and lead citrate. Microscopy was carried out using a Zeiss 900 transmission electron microscope operated at 80 kV. Several electron micrographs were recorded digitally at ×3000 for each ultrathin nerve section.

### 2.6. Immunoperoxidase Labeling

Sections were incubated for 10 min with 0.3% H_2_O_2_ in methanol then washed three times in 0.1 M PBS (pH 7.4). To block nonspecific sites, sections were incubated in PBS containing 0.3% Triton X-100 and 10% normal donkey serum at room temperature for 1 h, then incubated overnight in primary antibodies (goat polyclonal anti-Sema3D, 1 : 100, Santa Cruz, sc-67943; rabbit polyclonal anti-Plexin, 1 : 100, Santa Cruz; and mouse polyclonal anti-Neuropilin1, 1 : 100, Abcan, AB81321). Slides were washed three times in PBS, incubated in the appropriate secondary antibodies (biotinylated horse anti-rabbit; horse anti-goat and goat anti-mouse; 1 : 500 dilution; Vector Laboratories, Burlingame, CA) for 2 h at room temperature, and then received ABC reagent (1 : 200 dilution in PBS) treatment (Vectastain Elite ABC kit, Vector, Switzerland). Color reaction used diaminobenzidine (DAB) substrate (Sigma) at room temperature. Slides were coverslipped using fluoromount (Sigma).

Quantification of immunoperoxidase data was performed using ImageJ [18] by determining immunostained densities. Three fields at 20x magnification at 1.5 mm from the injury site in longitudinal sections of the nerve were analyzed, and an average of the optical density was obtained. It used five animals per group.

### 2.7. Adult Dissociated Retinal Cell Culture

Retinal cultures from mice were prepared as described previously [19]. In brief, retinae were dissected from eyecups and digested in DMEM containing papain (10 U/mL, Worthington) and L-cysteine (0.2 mg/mL, Sigma) at 37°C for 30 minutes. Retinae were then triturated and washed by centrifugation in 50 mL DMEM (7 min at 500 g). Retinal pellets were resuspended in DMEM F12 containing B27-supplement (1 : 50), 0.25 *μ*g/mL fungizone, 100 *μ*g/mL penicillin/streptomycin, and methylcellulose (1 : 250). Cells were seeded into 24-well plates coated with poly-D-lysine (0.1 mg/mL, Sigma) and laminin (20 *μ*g/mL, Sigma). After 3 days in culture, cells were fixed in 4% PFA for 1 h. To identify RGCs and search for PlexinA1 receptor, cells were washed in PBS, incubated in blocking solution, and then in the TUJ-1 (1 : 200; BioLegend) and Plexin A1 primary antibodies (1 : 100, Santa Cruz). Sections were washed and incubated in appropriate secondary antibody (Alexa-fluor 488 Donkey anti-mouse; Alexa-fluor 594 Donkey anti-rabbit). Nuclei were stained with DAPI (Molecular Probes, USA) and mounted with fluoromount (Sigma).

### 2.8. Retinal Explant Culture

Retinal explant culture was performed as described previously [20]. Briefly, sterile laminules were placed in cell culture plates overnight with 0.3 mL of a 0.25 mg/mL poly-L-lysin (poly-L-lysine hydrobromide, P1274; Sigma, St. Louis, MO) solution in sterile PBS. The wells were rinsed twice with PBS and subsequently coated with 0.3 mL of a 2 *μ*g/mL laminin (Sigma) solution for 2 h and rinsed twice with PBS. Retinal explants were obtained from eye dissection in sterile CMF solution and placed in the wells with the RGC nerve fiber layer contacting the laminule, in 0.5 mL Neurobasal-A medium (Invitrogen), containing 0.4% methylcellulose (Sigma), 1% penicillin/streptomycin, 0.2% fungizone, 0.5% L-glutamine, and 2% B-27 supplement for retina (all from Invitrogen). The explants were cultured at 37°C with 5% CO_2_ for 72 h. Daily, 0.25 mL of the explant medium was replaced with fresh medium. Explants were washed in PBS, incubated in blocking solution, and then in the TUJ-1 (1 : 200; BioLegend) and Plexin A1 primary antibodies (1 : 100, Santa Cruz). Sections were washed and incubated in appropriate secondary antibody (Alexa-fluor 488 Donkey anti-mouse; Alexa-fluor 594 Donkey anti-rabbit). Nuclei were stained with DAPI (Molecular Probes, USA) and mounted with fluoromount (Sigma).

### 2.9. Statistical Analysis

Statistical analyses graphs were plotted using GraphPad Prism 5.0 software, and the statistical analysis was performed using unpaired *t*-test, and differences were considered significant when ^∗^*P* ≤ 0.05, ^∗∗^*P* ≤ 0.01, and ^∗∗∗^*P* ≤ 0.001.

## 3. Results

### 3.1. Optic Nerve Regeneration

**Figure 1:**
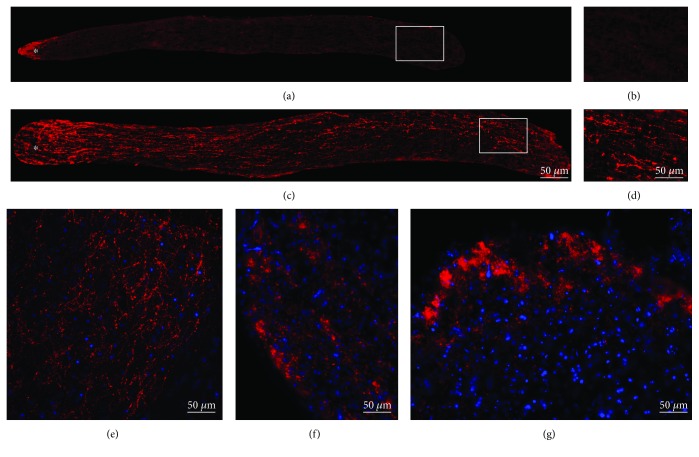
Optic nerve regeneration. (a–d) CTB labeling (red) and nuclear staining (blue) two weeks after optic nerve injury; (a and b) optic nerve from the Crush-only group exhibiting no CTB^+^ axons (red) beyond injury site; (c and d) optic nerve from the Regenerating group showing full-length axon regeneration; (e–g) CTB (red) and nuclear labeling (blue) showing different levels of reinnervation of the visual nuclei twelve weeks after optic nerve injury. (e) CTB^+^ axons at the suprachiasmatic nucleus; (f) CTB^+^ fibers at the lateral geniculate nucleus; (g) CTB^+^ axons at the superior colliculus. *N* = 5 per group. (a–g) Scale bars: 50 *μ*m.

To confirm that the treatment was able to regenerate axons along the optic nerve, we injected CTB to trace retinal projections. After two weeks, it was already possible to visualize differences between the groups. The untreated group (Crush-only) showed no axons beyond the injury site. Instead, as expected, treated group (Regenerating) exhibited numerous CTB^+^ axons extending the full length of the optic nerve (Figures 1(a)–1(d)). At two weeks after injury and treatment, few axons from the Regenerating group were able to penetrate the chiasm and there were no visible axons crossing this structure.

To search for central projections, animals were left in a longer survival time. Twelve weeks after injury and treatment, animals from the Regenerating group had axons regenerating into appropriate target areas. The contralateral suprachiasmatic nucleus (SCN) exhibited many regenerating axons (Figure 1(e)), and sometimes the projections were bilateral (data not shown). The dorsal lateral geniculate nucleus (dLGN), in the contralateral side to the regenerating nerve, showed a stronger CTB labeling (Figure 1(f)). The contralateral superior colliculus (SC) also exhibited many CTB^+^ fibers (Figure 1(g)). CTB^+^ fibers were never seen in the Crush-only group.

### 3.2. Myelin Content within the Optic Nerve

**Figure 2:**
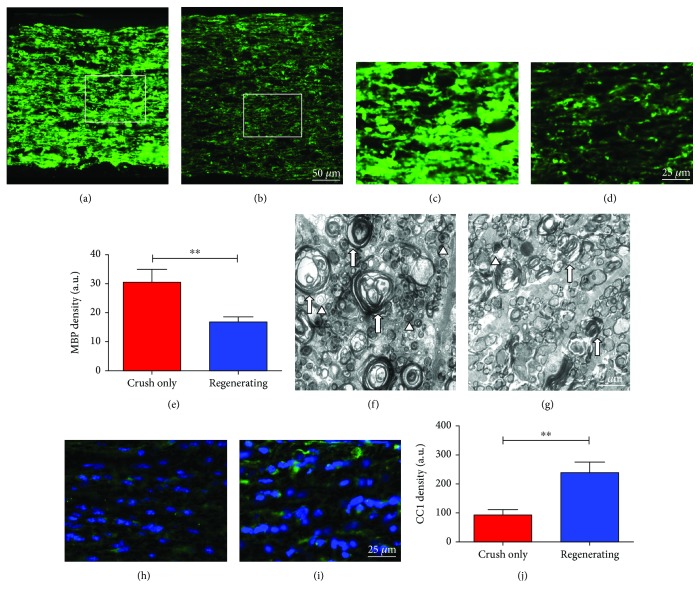
Myelin content within the optic nerve. (a–d) MBP labeling (green) two weeks after optic nerve injury. (a and c) Optic nerve from the Crush-only group exhibiting large amount of MBP staining compared to (b and c) optic nerve from the Regenerating group. (e) Quantification of MBP levels. (f, g) EM images showing myelin debris in both groups. Arrows point to myelin debris, whereas arrowheads indicate degenerating nerve fibers; (h–j) CC1 staining (green) two weeks after optic nerve injury; (h) optic nerve from the Crush-only group exhibiting a reduced CC1 staining; (i, j) optic nerve image and quantitative analysis showing a higher density of CC1 staining in the Regenerating group. *N* = 5 per group. Scale bars: (a, b) 50 *μ*m; (c, d) 25 *μ*m; (f, g) 2 *μ*m; (h, i) 25 *μ*m. Error bars in (e) and (j) show SEM. ^∗∗^*P* < 0.01.

To visualize myelin content two weeks after injury, we assessed the major myelin protein, myelin basic protein, in both groups (Figures 2(a)–2(d)). The Regenerating group exhibited reduced levels of MBP reactivity compared to the Crush-only group, and our findings were confirmed by the quantification of MBP staining density in the analyzed tissues (Figure 2(e)). Qualitative analysis of electron microscope images also revealed that the Crush-only group showed more myelin debris (arrows) and axons at various stages of degeneration (arrowheads) when compared to the Regenerating group (Figures 2(f)–2(g)).

In order to investigate whether myelin content reduction was due to a reduced oligodendrocyte staining intensity and/or area, we used the oligodendrocyte marker CC1. However, the quantification showed that the CC1 staining was stronger in the Regenerating group compared to the Crush-only group (Figures 2(h)–2(j)), suggesting that the regenerating axons are possibly being myelinated.

### 3.3. Microglia/Macrophage within the Optic Nerve

**Figure 3:**
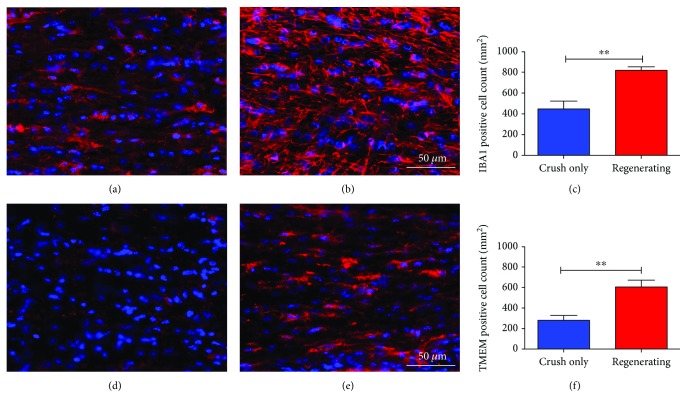
Macrophage/microglia within the optic nerve. (a–c) Iba-1 (red) and nuclear staining (blue) two weeks after injury. (a) Optic nerve from the Crush-only group showing a reduced Iba-1 staining; (b and c) optic nerve and quantitative analysis exhibiting intense levels of Iba-1 in the Regenerating group. (d–f) Tmem (red) and nuclear (blue) staining two weeks after injury. (d) Optic nerve from the Crush-only group. (e and f) Optic nerve image and quantitative analysis presenting higher levels of Tmem density in the Regenerating group. *N* = 5 per group. Scale bars: (a, b, d, e) 50 *μ*m. Error bars in (c) and (f) show SEM. ^∗∗^*P* < 0.01.

Since microglia and macrophages are the cells responsible for the myelin clearance, we decided to investigate the number of these cells in the optic nerves. The Regenerating group exhibited a higher number of Iba-1-positive cells compared to the Crush-only group, and our findings were confirmed by quantification of Iba-1-positive cells in the analyzed tissues (Figures 3(a)–3(c)). In order to visualize only the microglia content, we used a specific microglia marker called TMEM119. The Regenerating group presented an increase in the number of TMEM119-positive cells, when compared to the Crush-only group (Figures 3(d)–3(f)).

### 3.4. Reactive Astrogliosis within the Optic Nerve

**Figure 4:**
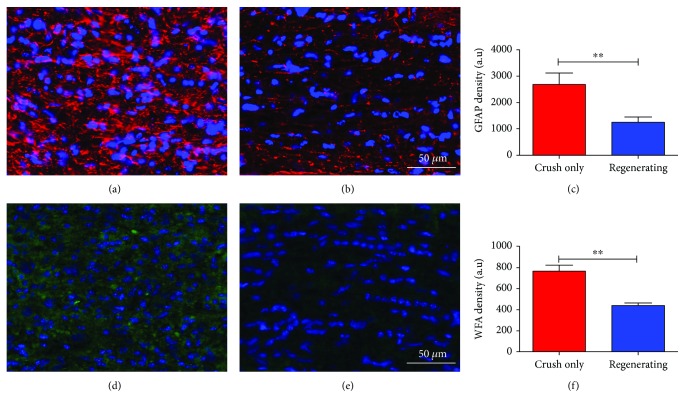
Reactive astrogliosis within the optic nerve. (a–c) GFAP (red) and nuclear staining (blue) two weeks after injury. (a) Optic nerve from the Crush-only group showing an intense GFAP staining; (b and c) optic nerve and quantitative analysis exhibiting reduced levels of GFAP in the Regenerating group. (d–f) WFA (green) and nuclear staining (blue) two weeks after injury. (d) Optic nerve from the Crush-only group. (e and f) Optic nerve image and quantitative analysis presenting reduced levels of WFA density in the Regenerating group. *N* = 5 per group. Scale bars: (a, b, d, e) 50 *μ*m. Error bars in (c) and (f) show SEM. ^∗∗^*P* < 0.01.

Knowing that one of the inhibitory signs of axonal growth, namely, myelin debris, had been softened after the treatment, we decided to investigate other repulsive signs to axonal regeneration in the central nervous system. Reactive astrogliosis is considered another major inhibitory signal to axonal growth. Following CNS injuries, astrocytes around the injury site are converted into reactive astrocytes and eventually into scar-forming astrocytes that block axon regeneration and neural repair [21]. So, we investigated by means of GFAP immunofluorescence, if the treatment changed the astrocytic reaction after optic nerve injury. Indeed, the Regenerating group presented a decrease in GFAP immunoreactivity (Figures 4(a)–4(c)), when compared to the Crush-only group. As a second step in this process, we examined the expression of CSPG in the optic nerve by staining for *Wisteria floribunda* agglutinin (WFA), a plant lectin reported to selectively bind to N-acetylgalactosamine residues, associated with chondroitin sulfates (CSs) within the extracellular matrix. The Regenerating group presented a decrease in WFA staining (Figures 4(d)–4(f)), when compared to the Crush-only group.

### 3.5. Semaphorin Signaling within the Optic Nerve

**Figure 5:**
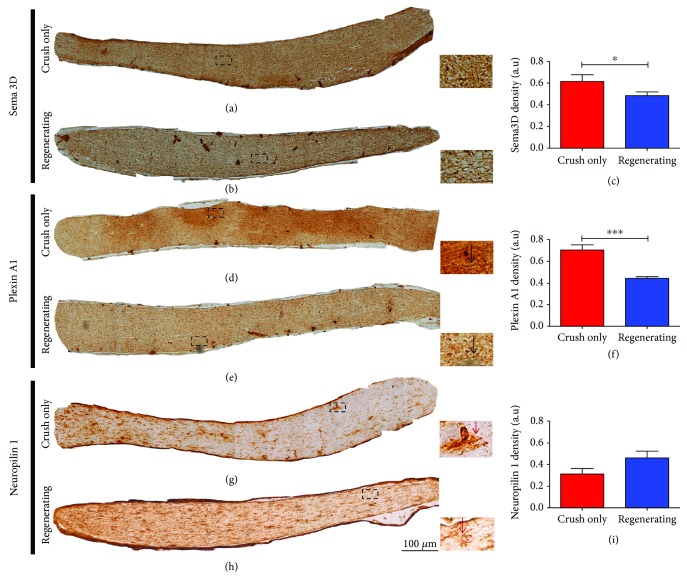
Semaphorin signaling within the optic nerve. (a) Optic nerve from the Crush-only group and inset representing the square region; (b) optic nerve from the Regenerating group and inset representing the square region; (c) quantitative analysis of semaphorin staining. (d) Optic nerve from the Crush-only group and inset representing the square region; (e) optic nerve from the Regenerating group and inset representing the square region; (f) quantitative analysis of Plexin A1 staining. (g) Optic nerve from the Crush-only group and inset representing the square region; (h) optic nerve from the Regenerating group and inset representing the square region; (i) quantitative analysis of Neuropilin 1 staining. All analyses were done in the survival time of two weeks after injury. *N* = 5 per group. Scale bars: 100 *μ*m. Error bars in (c, f, i) show SEM ^∗^*P* < 0.05 and ^∗∗∗^*P* < 0.001.

Secreted class 3 semaphorins (Sema3s) are also described as repulsive signals which are present after CNS injury [22–24]. We chose to investigate the role of Sema3D and its receptors Plexin A1 and Neuropilin 1. Sema3D is a semaphorin of unknown function during the process of mammal CNS degeneration and regeneration. The immunoreaction for Sema3D showed a diffuse pattern characteristic of secreted molecules. Our results showed a reduction in Sema3D expression on the optic nerves from the Regenerating group compared to the Crush-only group (Figures 5(a)–5(c) and insets). The receptor PlexinA1 also exhibited a smaller density in the Regenerating group compared to the Crush-only group, in a pattern similar to nerve fibers (Figures 5(d)–5(f); black arrows in the insets). However, the Neuropilin 1 receptor showed no difference between groups, presenting a staining pattern that suggest glial cell bodies and its proximal processes (Figures 5(g)–5(i) and red arrows in the insets). Thus, in addition to the reduction in the expression of the inhibitory molecule Sema3D (Figures 5(a)–5(c)), there was also a reduction in the expression of the receptor Plexin A1 (Figures 5(d)–5(f)).

**Figure 6:**
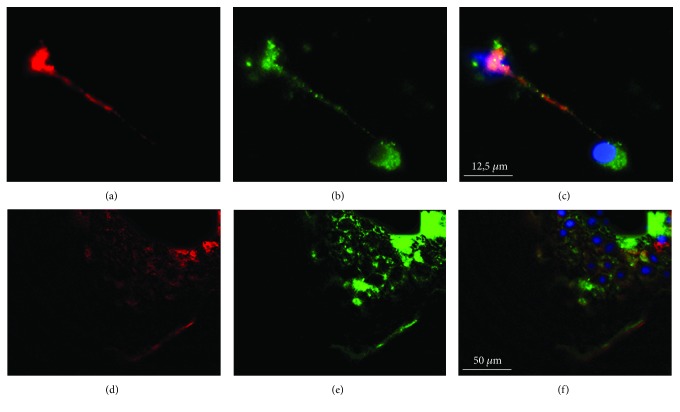
*In vitro* expression of Sema3D receptors. (a, b, c) Retinal dissociated culture expressing the receptor Plexin A1 (red) in Tuj-1-positive cells (green) and nuclear staining (DAPI). (d, e, f) Retinal explant cells expressing the receptor Plexina A1 in Tuj-1-positive cells. *N* = 3 per group. Scale bars: (a, b, c) 12.5 *μ*m, (d, e, f) 50 *μ*m.

Since the in vivo staining of Plexin A1 did not lead to certainty that regenerating nerve fibers were Plexin A1-positive within their navigating axons, we used different *in vitro* assays to access it. Analyses of the immunocytochemistry in the retinal dissociated culture and in retinal explants from animals submitted to the same treatment (PTEN gene deletion within retinal ganglion cells, associated to zymosan and cAMP intravitreous injection) indicated that they do express the receptor Plexin A1 in Tuj-1-positive neurites and cell bodies (Figures 6(a)–6(f)).

## 4. Discussion

Even after stimulation of the intrinsic growth properties of the optic nerve, most regenerating axons are stuck proximal to the glial scar that is formed within the lesion site [25]. Axons that overcome this barrier still undergo U-turns or are incorrectly guided to the wrong axon tract or to the contralateral optic nerve [15, 26, 27]. These findings pointed out the importance of investigating the environmental molecules that drive growth cone guidance during adult CNS regeneration. Even with these barriers, different treatments described full-length optic nerve regeneration, with target reinnervation, synapse formation, and partial function recovery [11, 12, 28]. In the present study, we investigated the effect of the conditional deletion of PTEN in RGC of mice submitted to intraocular inflammation + cAMP during optic nerve regeneration on the content of the repulsive axon guidance cues Sema3D and its receptors, myelin debris, and astrogliosis, within the axon shaft distal to the lesion site.

Our results were able to replicate the data provided by de Lima et al. [12], who found that conditional deletion of PTEN in RGCs followed by intraocular injection of zymosan and cyclic AMP promoted full-length regeneration of optic nerve axons within 2 weeks, reaching SCN, dLGN, and SC, 12 weeks after lesion. On the other hand, Luo et al. [27], using the same stimulatory protocol, argued that they found reinnervation of SCN only. It is possible that the whole-brain 3D imaging employed by Luo et al. [27], although presenting the advantage of allowing the tracing of the path followed by single CTB-traced fascicles, may be less sensitive than the microscopy of thin brain sections employed in the present study and in the study of de Lima et al. [12].

Interestingly, the research that focused in promoting the intrinsic growth capacity of RGC did not investigate if the microenvironment undergoes any modulation in molecules that promotes or inhibits axon regeneration. It is well known that Wallerian degeneration (WD) in the CNS is an inefficient process, since the removal of axon and myelin debris by microglia/macrophages is slow, leaving inhibitory molecules for long period of time, therefore blocking regeneration [29, 30]. In the injured peripheral nervous system (PNS), however, WD takes place in a more effective way resulting in a more efficient nerve regeneration. Accordingly, previous findings of our group [31, 32], in a model of peripheral nerve injury, showed that Galectin-3 knockout mice present earlier sciatic nerve regeneration due to a greater efficiency in the removal of myelin debris by macrophages and Schwann cells favoring an increase in WD speed. Therefore, it is plausible to say that in the present work the reduction in the amount of myelin debris is correlated with the better regeneration found in the treated group. In addition, we analyzed the oligodendrocyte staining and found an increase in CC1 density in the treated group, which corroborates our hypothesis that remyelination is occurring. In fact, this increase in oligodendrocyte staining might be responsible for myelination of regenerating fibers, as suggested by the excitable domain reorganization found after employment of the same protocol by Marin and coworkers [33]. Besides, we observed a higher microglia/macrophage count in the Regenerating group. This increase in phagocytes might be responsible for the better myelin clearance found within this group. Corroborating this idea, Stark and collaborators (2018) recently showed that inflammatory stimulation of ON regeneration promotes an increase in Iba-1 staining, which was correlated to myelin clearance, 7 days after lesion, within the lesion site itself. Considering that all microglia express Iba-1, our results show that most of the IBA-1-positive cells are microglia, suggesting a pivotal role for microglia in myelin clearance outside the lesion site. Contrary to our findings, Hilla and coworkers [34] showed that microglia was irrelevant in promoting optic nerve regeneration after inflammatory stimulus, although microglia depletion delayed the astrocytic repopulation of the crush site. So, further studies are required to fully address the responsible actors of myelin clearance and its role during optic nerve regeneration.

Astrocyte reaction has long been identified as a major event that occurs after CNS lesion that prevents axon passage through the secretion of inhibitory CSPG [35]. Accordingly, we found that the Regenerating group presented less GFAP immunostaining within the optic nerve when compared to the Crush-only group. Besides, *Wisteria floribunda* agglutinin staining revealed less CSPG within the Regenerating group. Corroborating our data, Luo et al. [27] showed that GFAP staining was more prevalent near the lesion site, an area that presented more frequent U-turns of regenerated fascicles after PTEN/SOCS3 conditional deletion in RGC. However, employing only inflammatory stimulation to induce RGC regeneration, Stark and coworkers (2018) found no modulation of GFAP within the lesion site 7 days after lesion. These findings suggest that the reduced astrogliosis observed in our Regenerating group might have allowed the proper regeneration of fibers that otherwise would be trapped into CSPG nets. Additionally, different protocols of induction of intrinsic growth properties of central axons might elicit different environmental responses.

Since semaphorins are the main molecules known to guide axons within the optic nerve during development, where it builds repulsive walls that confine travelling axons within the optic nerve [36], we investigated their signaling during adult optic nerve regeneration. Family 3 semaphorins correspond to microglia/macrophage, fibroblast, or Schwann cell secreted proteins that bind to the extracellular matrix, where it exerts its repulsive effects onto Plexin and Neuropilin receptors located in growth cones [37]. De Winter et al. [24] showed that several Sema3 family members are upregulated after spinal cord lesion. Nevertheless, SEMA 3D is a secreted member of the SEMA family that was never investigated following mammal SNC lesions. Our results show that Sema3D content is higher in the Crush-only group when compared to the Regenerating group. Besides, Neuropilin 1 receptor was not altered by treatment, while Plexin A1 receptor was also higher in the Crush-only group, where it could signal the collapse of axons [38]. Correspondingly, Plexin A1 staining pattern was putative of nerve fibers, whereas neuropilin 1 staining pattern was similar to soma and proximal processes of putative glial cells. Thus, plexin A1 might be responsible for RGC navigation control during optic nerve regeneration, while Neuropilin1 may not be involved in this process. Since there is no study addressing if adult mice RGCs are able to respond to semaphorins and the immunohistochemistry was not sufficient to prove that RGC axons exhibited Sema3 receptors, we employed explant and dissociated retinal cultures that confirmed that adult RGC extending neurites present Plexin A1, therefore being sensitive to Sema3D levels. These results suggest for the first time that Sema3D may be another inhibitory molecule of axonal regeneration in rodent CNS, which could signal through Plexin A1. A repulsive role of Sema3D is consistent with previous works in which RGC growth cones of zebrafish were repelled by Sema3D [39]. Similarly, pharmacological inhibition of Sema3A leads to spinal cord regeneration and protects RGC from axon transection-induced apoptosis [40, 41]. Additionally, chondroitinase ABC treatment reduces Sema3s binding to the extracellular matrix, favoring regeneration (Soleman et al., 2002). Although our and other groups' results suggest that at least a subset of injured adult CNS neurons is inhibited by Sema3D or other Sema3s, future studies employing genetically manipulated mice or other means of blocking semaphorin receptor or intracellular signaling will be required to show without doubt that injured neurons sense and respond to semaphorins following optic nerve injury in vivo.

How PTEN conditional deletion on RGC followed by intravitreal injection of zymosan and cyclic AMP modifies the environment and aids ON regeneration is not currently known. It is possible that zymosan injection might be responsible for the higher proportion of microglia/macrophage number found in our study. Besides, growth cone protrusions, called invadosomes, present metalloproteinases that degrades the extracellular matrix [42] possibly reducing CSPG content and Sema3D content. Corroborating this line of evidence, neuroblastoma-derived growth cones secrete tissue plasminogen activator which activates the serine protease plasminogen, which also degrades extracellular matrix [43].

## 5. Conclusion

Together, our results provide, for the first time, evidence that several regenerative repulsive signals are reduced in regenerating optic nerve fibers following a combined therapy. Therefore, the treatment used was not only capable to stimulate the intrinsic growth capacity of RGCs but also made the CNS microenvironment more permissive to regeneration.

## Data Availability

The data used to support the findings of this study are available from the corresponding author upon request.
